# Effect of Ivabradine on Heart Failure: A 2024 Meta-Analysis

**DOI:** 10.7759/cureus.77346

**Published:** 2025-01-12

**Authors:** Akshay Maharaj, Matthew B Maturasingh, Alvin Khangembam, Sajay N Bidhesi, Shanzey Rai, Kamille A Garness, Nick Khadoo, Nimish Tutwala, Natalia A Khan, Jonelle J Ramsarran, Amit Bhandari, Pranaya Rajbhandari, Khin Linn Htet, Mohammad M Husain, Sinead N Bhagwandeen, Keston Rattan

**Affiliations:** 1 Internal Medicine, Port of Spain General Hospital, Chaguanas, TTO; 2 Critical Care Medicine, University of the West Indies at St. Augustine, St. Augustine, TTO; 3 Internal Medicine, North Eastern Indira Gandhi Regional Institute of Health & Medical Sciences (NEIGRIHMS), Shillong, IND; 4 Internal Medicine, San Fernando General Hospital, Couva, TTO; 5 Internal Medicine, Islamic International Medical College, Islamabad, PAK; 6 Biology, Saint Leo University, Saint Leo, USA; 7 Global Health, George Washington University, Washington, USA; 8 Department of Medicine, Eric Williams Medical Sciences Complex, Mt. Hope, TTO; 9 Obstetrics and Gynaecology, Topiwala National Medical College & B. Y. L. Nair Charitable Hospital, Mumbai, IND; 10 Internal Medicine, Fazaia Medical College, Islamabad, PAK; 11 Obstetrics and Gynaecology, Mount Hope Women's Hospital, Valsayn, TTO; 12 Internal Medicine, American University of the Caribbean School of Medicine, Cupecoy, SXM; 13 Internal Medicine, Nepal Medical College, Kathmandu, NPL; 14 Family Medicine, University of Medicine 2, Yangon, Yangon, MMR; 15 Internal Medicine, Gulf Coast Medical Center, Port Richey, USA; 16 Internal Medicine, Howard University Hospital, Washington, USA; 17 Internal Medicine, State University of New York Downstate Medical Center, New York City, USA

**Keywords:** article review, heart failure with preserved ejection fraction, heart failure with reduced ejection fraction, ivabradine, left ventricular ejection fraction (lvef), pharmacotherapy for heart failure

## Abstract

Ivabradine is thought to be highly effective in the treatment of heart failure through its effects on heart rate (HR) and left ventricular ejection fraction (LVEF). However, its effectiveness on other clinical outcomes and cardiovascular parameters is undetermined. Therefore, a meta-analysis of existing clinical studies was conducted to determine the effectiveness of ivabradine in treating chronic heart failure. A primary search was employed using five databases, namely, PubMed, Medline, Clinical Trials, Embase, and Cochrane. A meta-analysis was conducted on 11 studies meeting the inclusion criterion, including randomized control trials of chronic heart failure patients receiving ivabradine versus standard care or placebo treatment. Non-human studies and studies without echocardiogram measures of ejection fraction, a placebo group, or follow-up data on readmission or mortality were restricted from the study; however, there were no restrictions on the date or duration of treatment. The outcomes measuring the effectiveness of the drug included cardiovascular mortality, hospital readmissions, and exercise capacity, in addition to changes in HR, LVEF, Minnesota Living with Heart Failure (MLWHF) questionnaire, N-terminal pro-hormone of brain natriuretic peptide (NT-proBNP), brain natriuretic peptide (BNP) levels, and left ventricular volume. The reduction in bradycardia and atrial fibrillation was also determined. Subsequently, data from 11 randomized clinical trials including 1,687 study participants, 862 in the ivabradine treatment group, and 825 in the placebo group were included in the analysis. No significant reduction in cardiovascular mortality or hospital readmissions was noted with the use of ivabradine compared to the placebo group (relative risk (RR)), 0.79 (95% confidence interval (CI)), 0.15, 4.14) and (RR, 0.53 (95 % CI, 0.20, 1.22), respectively. In 10 of these trials, there was a significant reduction in the HR of treatment participants (mean difference (MD)), - 11.7 (95 % CI, -12.88, -10.51). There was also a beneficial association noted between LVEF and ivabradine participants (MD, 3.03 (95 % CI, 2.07, 3.98). There was a significant reduction in NT-proBNP levels (MD, -384 (95 % CI, -581.68, -187.72) in ivabradine patients, but no significant change was noted in BNP levels in this group (MD, -72.32; 95 % CI, -263.67, 119.0) The risk reduction in bradycardia and atrial fibrillation among ivabradine users versus non-users were both insignificant (RR, 1.62 (95 % CI, 0.024, 4.83) and RR, 0.93 (95 % CI, 0.014, 12.51), respectively). Conclusively, heart failure patients taking ivabradine demonstrated significant improvements in LVEF and reduction in HRs compared to the standard treatment group. No significant changes in other cardiovascular or clinical outcomes in Ivabradine users were confirmed in this meta-analysis.

## Introduction and background

Chronic heart failure is a common but complex progressive cardiovascular syndrome that could result from any cardiovascular disease (CVD) or condition like coronary heart disease, cardiomyopathies, and congenital cardiac condition, which affects the functioning of the left ventricle [1]. Left ventricular ejection fraction (LVEF) is an important indicator of the underlying pathophysiology of the disease and its sensitivity to treatment. Hence, LVEF is used to classify heart failure into heart failure with preserved ejection fraction (HFpEF), LVEF 40-49%, and heart failure with reduced ejection fraction (HFrEF), LVEF <40% [2]. HFrEF is considered systolic heart failure resulting from the impairment of left ventricular function, whereas HFpEF is considered diastolic heart failure. However, any type of heart failure is associated with varying degrees of structural and functional remodeling of the heart tissue [1].

Heart rate (HR) is an important and independent risk factor for heart failure. Elevated HR causes an increase in oxygen demand, which may lead to the rupture of the coronary plaque, and also decreases the diastolic phase of coronary perfusion, thus causing myocardial ischemia, which can in turn cause severe adverse cardiac events leading to death [3]. A 14% increment in all-cause mortality with an increase in an HR of 10 beats per minute and a two-fold increase in incident heart failure was observed in the Framingham study [4]. Thus, studies have shown that maintaining an HR of 60 to 70 beats per minute is an important therapeutic goal in patients with heart failure [5].

Ivabradine is a pure HR-lowering agent that selectively inhibits the cardiac pacemaker by blocking the funny current channels in the sinoatrial node. However, it does not decrease the effect of cardiac contractility on blood pressure [3]. In 2015, the US Food and Drug Administration (FDA) officially approved ivabradine in the treatment of HFrEF in patients with an HR of more than 70 beats per minute on the maximum dose of beta blockers or in patients where beta blockers are contraindicated [5]. The European Society of Cardiology (ESC) guidelines also recommend the same [1]. Ivabradine, however, was seen to increase the relative risk of atrial fibrillation by 24%. In patients with HFpEF, ivabradine was seen to reduce the HR, but it did not affect left ventricular relaxation or filling pressure and hence had no effect on improving mortality in these patients [1]. Ivabradine can therefore be a promising drug for improving outcomes in patients with HFrEF; however, its safety and efficacy have to be further analyzed. A meta-analysis of six randomized controlled trials (RCTs) on the effect of ivabradine on HFrEF patients showed an enhanced improvement in cardiac function with improved exercise capacity. It also reduced worsening readmissions in heart failure patients. However, these studies also had their limitations, like the dose-effect relationship that could not be analyzed, and the heterogeneity and number of patients also affected the analysis [5]. Thus, more large-scale studies are warranted to prove the efficacy and safety of ivabradine in patients with heart failure. This is a meta-analytical study across different databases including 960 articles on the safety and efficacy of ivabradine in patients with heart failure so that we can have a safer alternative to beta blockers in heart failure patients to reduce the HR and improve the outcome.

## Review

This study was organized under the standard article protocol for Medical Journals, following the Preferred Reporting Items for Meta-Analysis (PRISMA) checklist. Robvis risk-of-bias domains were used to analyze the bias categories of each study [6].

Search strategy

**Figure 1:**
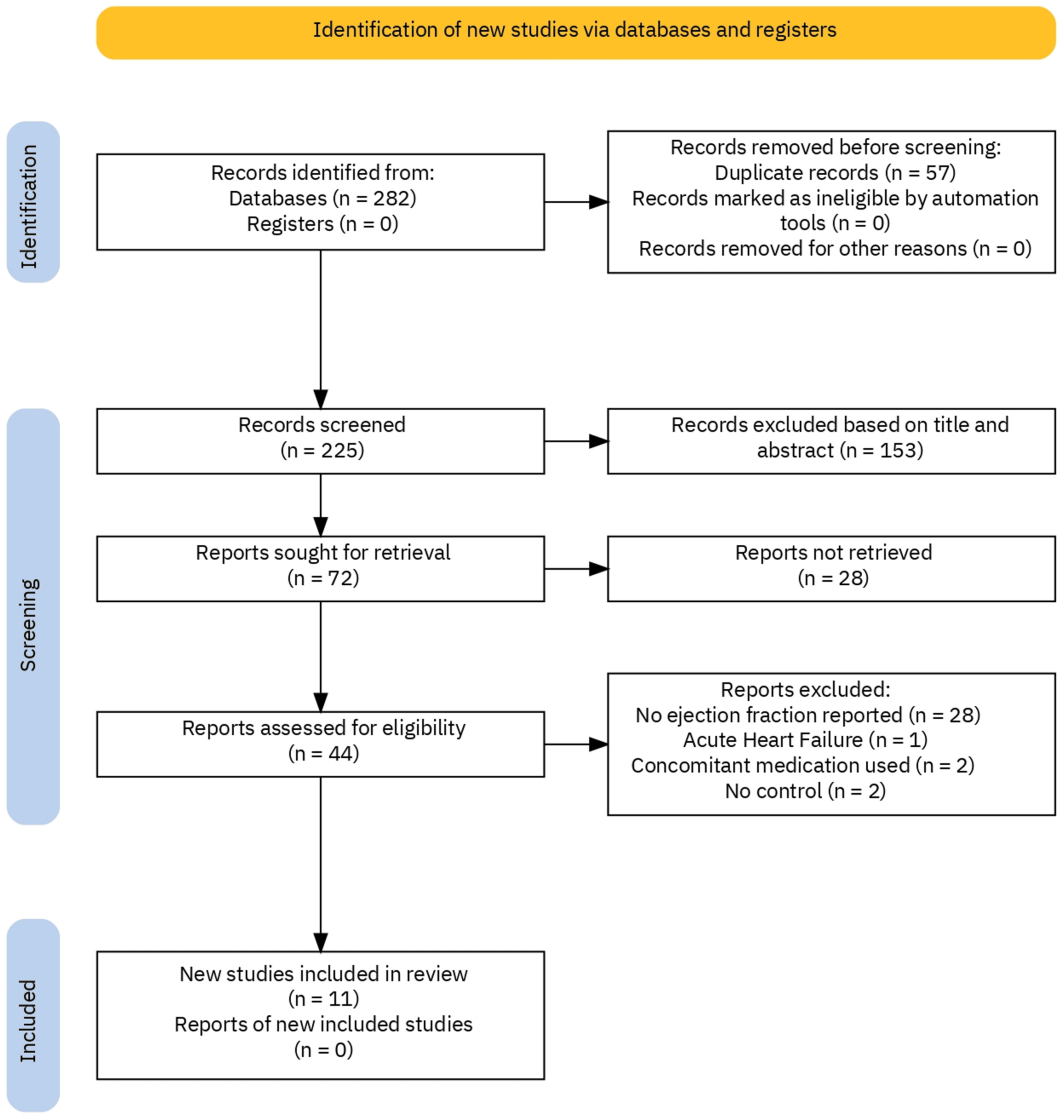
A stratified flow diagram of all studies identified, with screening in accordance with inclusion and exclusion criteria

Our team scanned PubMed, Medline, Clinical Trials, Embase, and the Cochrane Central Register of Controlled Trials using the search terms “ivabradine or Corlanor or Procoralan” as drug names and “heart failure” along with a filter for clinical trials without specific time restriction (Figure 1).

Inclusion/exclusion criteria

The following inclusion criteria were chosen: (1) control trials only comparing ivabradine to any placebo, such as beta-blockers, angiotensin-converting enzyme (ACE) inhibitors, angiotensin receptor blockers (ARBs), diuretics, and aldosterone antagonists); (2) heart failure of any type, heart failure with reduced ejection fraction (HFrEF) or heart failure with preserved ejection fraction (HFpEF); (3) studies that must include echocardiographs (ECHOs) to measure ejection fraction; (4) studies with follow-up data (cardiovascular mortality, re-admission); and (5) must be in English.

The following exclusion criteria were (1) non-human studies, (2) articles in a language other than English solely, (3) no echocardiograph/follow-up data, and (4) no comparison between intervention and control groups. The characteristics of the included studies are seen in Table 1.

**Table 1:** Characteristics of the included studies LVEF: left ventricular ejection fraction, LV: left ventricular, RCT: randomized controlled trial, n: number of participants, NYHA: New York Heart Association, HR: heart rate, HF: heart failure, HFpEF: heart failure with preserved ejection fraction, HFrEF: heart failure with reduced ejection fraction, DCM: dilated cardiomyopathy, CAD: coronary artery disease, SBP: systolic blood pressure

Study	Method	Participants	Intervention	Outcome	Duration
Bonnet et al., 2017 [7]	RCT	116 children were randomized to either ivabradine or placebo which was titrated to reach the primary endpoint. Clinical status (New York Heart Association or Ross status), LV function, pro-B-type natriuretic peptide, and quality of life were measured.	Single-dose units of 10 mL containing 1, 5, or 13.3 mg of ivabradine	Reduction of baseline resting HR and decreased LVEF	12 months
Ye et al., 2022 [8]	RCT	181 patients were assigned to placebo and 179 patients were assigned to ivabradine, all aged between 18-75 years old with EF < 40% and New York Heart Association class II-IV.	Ivabradine 5 mg once daily titrated to 15 mg	Improvement in LV end-systolic volume over 32 weeks	32 weeks
Tsutsui et al., 2019 [9]	RCT	26 patients in the ivabradine group and 37 patients in the placebo group were randomized, all >20 years old, and had symptomatic chronic HFrEF under New York Heart Association Class II-IV and LVEF <35%.	Ivabradine 2.5-7.5 twice daily	Composite of cardiovascular death or hospital admission for worsening HF	54 weeks: two weeks observation and 52 weeks treatment.
Dogheim et al., 2022 [10]	RCT	60 patients with systolic chronic HF for more than 4 weeks and LVEF < 35% and NYHA class II-IV randomized to ivabradine or placebo	Ivabradine 5 mg twice daily	There was a significant decrease in NYHA class and HR in the ivabradine group.	Three months
Raja et al., 2017 [11]	RCT	187 total patients with HF characterized by NYHA II-IV, DCM, and HR >70. 62 were excluded due to CAD, valvular disease, and atrial fibrillation. Of the remaining, 63 were assigned to the control group and 62 were given ivabradine.	Ivabradine 13.3 +/- 2.3 mg given once daily	Reduction of HR in patients with HF with DCM without difference in SBP	Six months
Tsutsui et al., 2016 [12]	RCT	126 stable Japanese patients with HFrEF, EF < 35%, HR > 75 beats/min, and NYHA Class II-IV divided into 3 groups: placebo, ivabradine 2.5 mg, and ivabradine 5 mg.	Ivabradine 2.5 mg twice daily	Significant decrease in NYHA class and HR in the ivabradine group	Six weeks
Tsutsui et al., 2016 [12]	RCT	126 stable Japanese patients with HFrEF, EF < 35%, HR > 75 beats/min, and NYHA Class II-IV divided into 3 groups: placebo, ivabradine 2.5 mg, and ivabradine 5 mg.	Ivabradine 5 mg twice daily	Significant decrease in NYHA class and HR in the ivabradine group	Six weeks
Kosmala et al., 2013 [13]	RCT	61 patients with HFpEF were randomly assigned in a double-blind study to the placebo or ivabradine group.	Ivabradine 5 mg twice daily	Increased exercise capacity and LV filling pressure in the ivabradine group	Seven days
Sarullo et al., 2010 [14]	RCT	60 patients with 13 more not deciding to participate. These 60 patients had LVEF <40% and NYHA class II-III.	Ivabradine 5 mg twice daily	Improvement in exercise capacity, gas exchange, and NYHA outcomes	Three months
Abdel-Salam et al., 2014 [15]	RCT	43 patients with DCM and LVEF <40%, NYHA >class II, and HR >70 randomized to get ivabradine (n=20) or placebo (n=23).	Ivabradine 7.5 mg twice daily	Improvement in functional capacity and reduction of HR	Three months
Mansour et al., 2011 [16]	RCT	167 patients with chronic HF screened for NYHA class III/IV and LVEF <40%. Of the total, 53 patients were chosen and 23 were assigned to standard placebo and 30 to ivabradine treatment.	Ivabradine was titrated from 2.5 mg once daily to 7.5 mg twice daily.	Significant reduction in HR with improvement in LVEF, LV volume, and NYHA symptoms	Three months

Primary and secondary outcomes

Each of the 11 studies was carefully analyzed to determine the primary outcomes of ivabradine on LVEF and HR, while also making sure to note secondary outcomes: quality of life (QoL), brain natriuretic peptide (BNP) levels, N-terminal pro-hormone of brain natriuretic peptide (NT-proBNP), exercise capacity, heart failure readmission, CVD mortality, asymptomatic bradycardia, and atrial fibrillation.

Data analysis and interpretation

Three teams of two investigators each were tasked with screening studies based on the aforementioned inclusion and exclusion criteria, using Covidence and RefWorks to eliminate duplicates and using subgroup analysis to exclude studies with less than three months of follow-up. After narrowing down to 11 studies, the effect size and standard error were input into the JASP (version 0.19.0, JASP Team, 2024) to create a forest plot using measures of heterogeneity with a 95% CI. Chi and degrees of freedom (df) were interpreted together, investigating the source of heterogeneity if Chi > df. Heterogeneity was measured by I^2^ with I^2^ < 50% and I^2^ > 50% needing further subgroup analysis to find the source of heterogeneity. Robvis was used to ascertain the level of bias among these studies.

Assessment of the risk of bias and quality of studies

**Figure 2:**
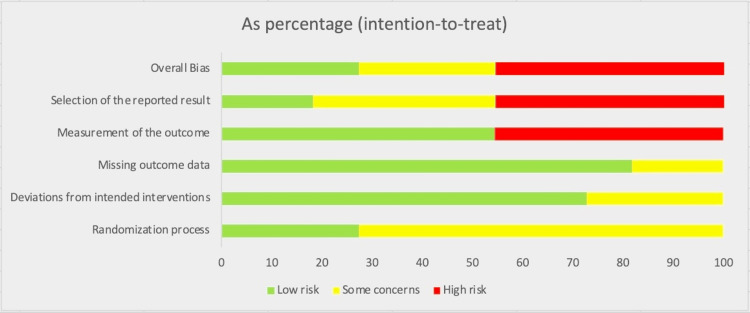
Bar graph showing the percentage risk of bias for each domain of the involved studies. The studies used to generate the risk of bias figure above include Bonnet et al. (2017) [7], Ye et al. (2022) [8], Tsutsui et al. (2019) [9], Dogheim et al. (2022) [10], Raja et al. (2017) [11], Tsutsui et al. (2016.1) [12], Tsutsui et al. (2016.2) [12], Kosmala et al. (2013) [13], Sarullo et al. (2010) [14], Abdel-Salam et al. (2014) [15], and Mansour et al. (2011) [16].

The Robvis risk-of-bias domains were used to analyze the bias categories of each study. Robs 2 classified each study into low, high, and some concern risk of bias. These results were obtained after each study was assessed with respect to domains including the randomization process, deviation from intended intervention, missing outcome data, measurement of the outcomes, and the selection of the reported results. The quality of evidence extracted by two independent investigators with subsequent discrepancy analysis was employed, which was then settled by a third investigator via reasoning among both independent investigators. A funnel plot drawn for HRs and LVEFs was symmetrical. Egger's test yielded p values < 0.05, indicating a low risk of bias. Figure 2 shows each domain and the corresponding classification of bias for the overall studies. For both the "missing outcome data" and "deviation from intended result" domains, the lowest risk of bias was observed. On the contrary, "measurement of outcome" and "selection of reported result" had the highest risk of bias.

**Figure 3:**
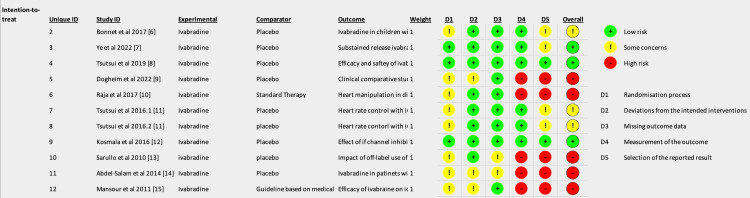
Individual studies' risk of bias for each domain and subsequent overall risk of bias. Studies included: Bonnet et al. (2017) [7], Ye et al. (2022) [8], Tsutsui et al. (2019) [9], Dogheim et al. (2022) [10], Raja et al. (2017) [11], Tsutsui et al. (2016.1) [12], Tsutsui et al. (2016.2) [12], Kosmala et al. (2013) [13], Sarullo et al. (2010) [14], Abdel-Salam et al. (2014) [15], and Mansour et al. (2011) [16].

As per Figure 3 (traffic light plot), all studies with a high risk of bias had high risks for domains 4 and 5. Those studies with low risks of bias had no major concerns for all domains. Studies with some concerns with respect to bias had concerns in at least more than one domain where the randomization process domain (D1) was the most prevalent.

Results

Eleven RCTs with 1,687 participants were enrolled in this meta-analysis with 862 patients in the ivabradine arm and 825 in the placebo arm. The outcomes of this study were the effects of ivabradine on HR, LVEF, QoL, NT-proBNP, BNP levels, and adverse events including CVD mortality, heart failure readmission, asymptomatic bradycardia, and atrial fibrillation.

Effect of Ivabradine on HR

The combined results of 10 RCTs (a total of 1,276 patients with 654 receiving ivabradine and 622 receiving placebo) reported a significant reduction in resting HR in patients receiving ivabradine (MD -11.7; 95% CI -12.88, -10.51). 

**Figure 4:**
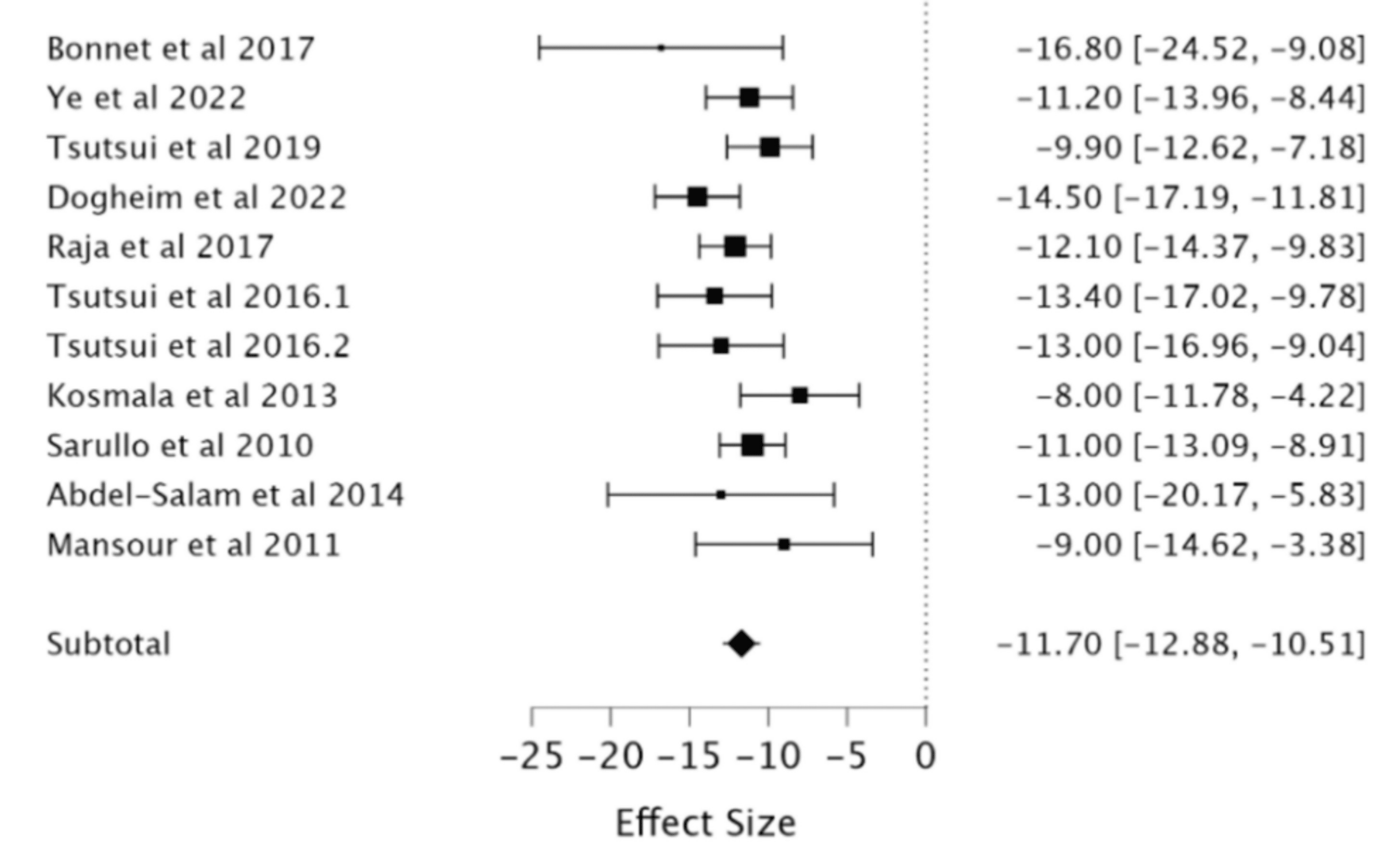
Forest plot of the mean difference in heart rate with ivabradine vs. placebo. Studies included: Bonnet et al. (2017) [7], Ye et al. (2022) [8], Tsutsui et al. (2019) [9], Dogheim et al. (2022) [10], Raja et al. (2017) [11], Tsutsui et al. (2016.1) [12], Tsutsui et al. (2016.2) [12], Kosmala et al. (2013) [13], Sarullo et al. (2010) [14], Abdel-Salam et al. (2014) [15], and Mansour et al. (2011) [16].

A sensitivity analysis, which excluded a study involving children in the population sample, maintained the pooled results (MD -11.58; 95%CI -12.77, -10.40) (Table 2, Figure 4).

**Table 2:** Effect of ivabradine vs. placebo on heart rate. HR: heart rate, SD: standard deviation, df: degree of freedom, P: p-value, I^2^ : Higgins I-squared statistic, CI: confidence interval

Study or subgroup	Mean HR Iva	SD HR Iva	Total Iva	Mean HR placebo	SD HR placebo	Total placebo	Mean difference	SE mean difference	Subtotal (95% CI)	Heterogeneity	Test of overall effect Z = -19.37
Bonnet et al., 2017 [7]	80.7	19.8	73	97.5	20.7	41	-16.8	3.939626234	-	-	-
Ye et al., 2022 [8]	69.5	13	170	80.7	13	172	-11.2	1.405943024	-	-	-
Tsutsui et al., 2019 [9]	66.7	11.4	127	76.6	10.7	127	-9.9	1.387372751	-	-	-
Dogheim et al., 2022 [10]	65.6	5.04	30	80.1	5.57	30	-14.5	1.371453001	-	-	-
Raja et al., 2017 [11]	63.8	3.6	63	75.9	8.4	62	-12.1	1.159214735	-	-	-
Tsutsui et al., 2016 [12]	66.4	7.2	41	79.8	9.4	41	-13.4	1.849192309	-	-	-
Tsutsui et al., 2016 [12]	66.8	8.8	40	79.8	9.4	41	-13	2.022652207	-	-	-
Kosmala et al., 2013 [13]	62	8	30	70	7	31	-8	1.927168517	-	-	-
Sarullo et al., 2010 [14]	63	3	30	74	5	30	-11	1.064581295	-	-	-
Abdel-Salam et al., 2014 [15]	68	11	20	81	13	23	-13	3.660304098	-	-	-
Mansour et al., 2011 [16]	72	13	30	81	7.7	23	-9	2.865512069	-	-	-
-	-	-	-	-	-	-	-	-	-11.7 [-12.88 , -10.51]	Chi² = 14.16	p= 0.001
-	-	-	-	-	-	-	-	-	-	df=10	-
-	-	-	-	-	-	-	-	-	-	P= 0.16	-
-	-	-	-	-	-	-	-	-	-	I² = 28.08	-

Effect of Ivabradine on LVEF

**Figure 5:**
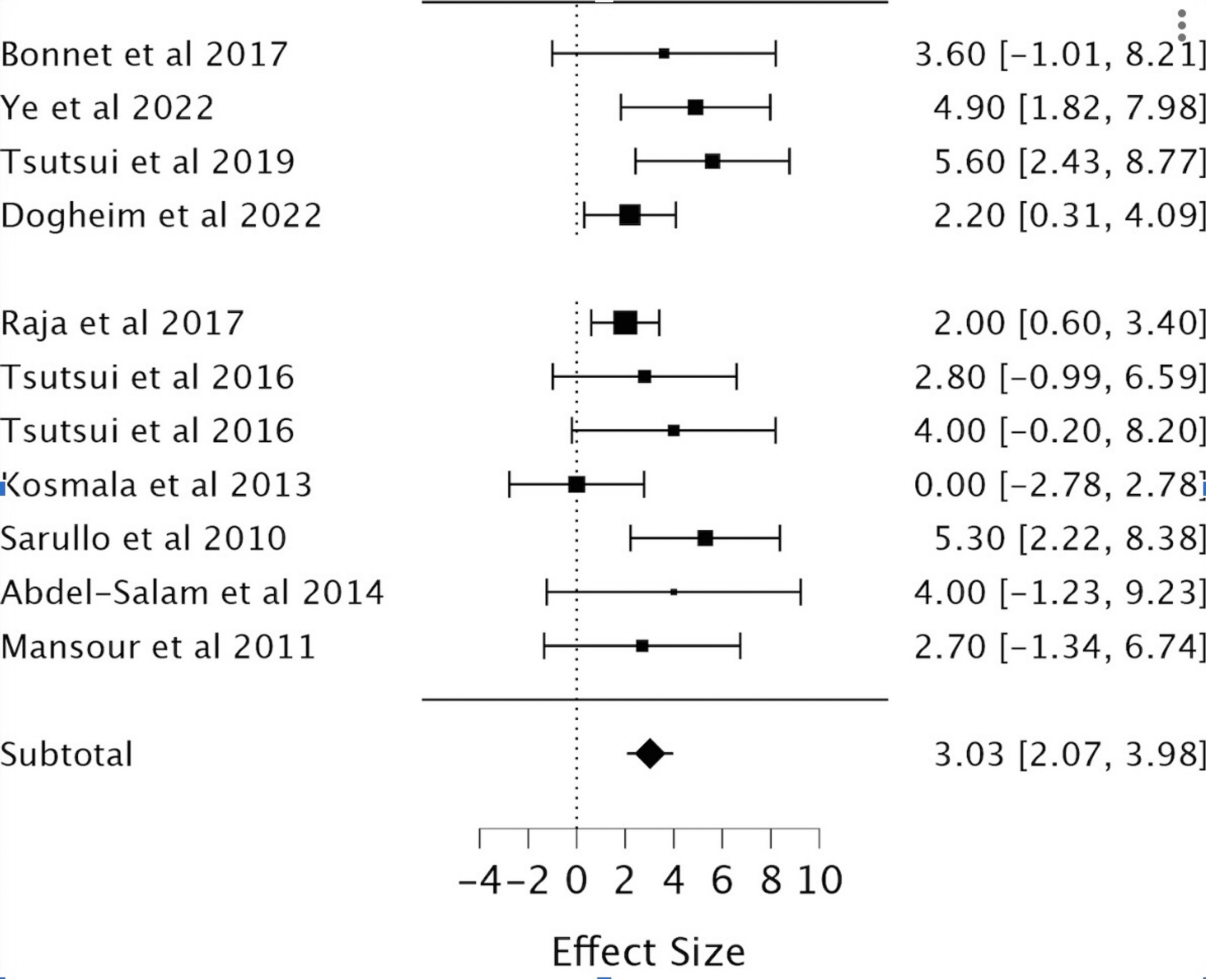
Forest plot of the mean difference in left ventricular ejection fraction (LVEF) with ovabradine vs. placebo. Sources include: Bonnet et al. (2017) [7], Ye et al. (2022) [8], Tsutsui et al. (2019) [9], Dogheim et al. (2022) [10], Raja et al. (2017) [11], Tsutsui et al. (2016.1) [12], Tsutsui et al. (2016.2) [12], Kosmala et al. (2013) [13], Sarullo et al. (2010) [14], Abdel-Salam et al. (2014) [15], and Mansour et al. (2011) [16].

The analysis of LVEF consisted of 11 RCTs (a total of 1,687 patients with 862 in the ivabradine group and 825 in the placebo), demonstrating a significant improvement in LVEF with the administration of ivabradine (MD 3.03; 95%CI 2.07, 3.98) (Table 3, Figure 5).

**Table 3:** Effect of ivabradine on LVEF compared to placebo. LVEF: left ventricular ejection fraction, SD: standard deviation, Iva: ivabradine, df: degree of freedom, P: p-value, I^2^: Higgins I-squared statistic, CI: confidence interval

Study or subgroup	Mean LVEF Iva	SD LVEF Iva	Total Iva	Mean LVEF placebo	SD LVEF placebo	Total placebo	Mean difference	SD mean difference	Subtotal (95% CI)	Heterogeneity	Test of overall effect Z=6.22
Bonnet et al., 2017 [7]	45.4	13.1	73	41.9	11.4	41	3.6	2.349591032	-	-	-
Ye et al., 2022 [8]	37.3	11.8	170	32.4	11.3	172	4.9	1.570562532	-	-	-
Tsutsui et al., 2019 [9]	38.9	12.8	127	33.3	13	127	5.6	1.618884617	-	-	-
Dogheim et al., 2022 [10]	29.77	4.11	30	27.57	3.31	30	2.2	0.9634694252	-	-	-
Raja et al., 2017 [11]	30.1	4	63	28.1	4	62	2	0.7155646512	-	-	-
Tsutsui et al., 2016 [12]	33.8	8.7	41	31	8.8	41	2.8	1.932583258	-	-	-
Tsutsui et al., 2016 [12]	35	10.4	40	31	8.8	41	4	2.143077341	-	-	-
Kosmala et al., 2013 [13]	68	6	30	68	5	31	0	1.416492715	-	-	-
Sarullo et al., 2010 [14]	35.4	5	30	30.1	7	30	5.3	1.570562532	-	-	-
Abdel-Salam et al., 2014 [15]	38	10	20	34	7	23	4	2.670287397	-	-	-
Mansour et al., 2011 [16]	36.8	8.3	30	34.1	6.7	23	2.7	2.061085264	-	-	-
-	-	-	-	-	-	-	-	-	3.03 [2.07, 3.98]	Chi² = 13.64	p= 0.001
-	-	-	-	-	-	-	-	-	-	df= 11	-
-	-	-	-	-	-	-	-	-	-	P= 0.25	-

Effect of Ivabradine on MLWHF-QoL

**Figure 6:**
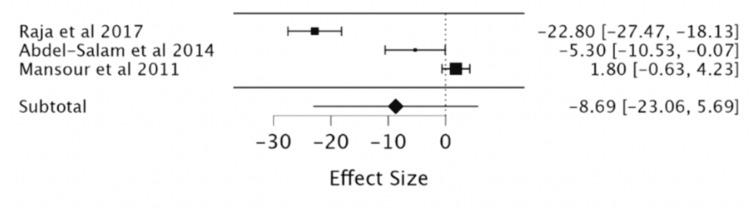
Forest plot of the mean difference in the MLWHF-QoL with ivabradine vs. placebo. Sources include: Raja et al. (2017) [11], Abdel-Salam et al. (2014) [15], and Mansour et al. (2011) [16].

Upon exploring the effect of ivabradine on the QoL of participants in three RCTs (a total of 221 patients, with 113 in the ivabradine group and 108 in the placebo group) using the Minnesota Living with Heart Failure Questionnaire (MLHFQ) questionnaire, no significant change was seen (MD -8.69; 95%CI -23.06, 5.69) (Table 4, Figure 6).

**Table 4:** Effect of ivabradine vs. placebo on MLWHF-QoL. QoL: quality of life, MLWHF: Minnesota Living with Heart Failure Questionnaire, SD: standard deviation, df: degree of freedom, P: P-value, I2 : Higgins I-squared statistic, CI: confidence interval

Study or subgroup	Mean MLWHF Iva	SD MLWHF Iva	Total Iva	Mean MLWHF placebo	SD MLWHF placebo	Total placebo	Mean difference	SD mean difference	Subtotal (95% CI)	Heterogeneity	Test of overall effect Z= -1.185
Raja et al., 2017 [11]	44.5	8	63	67.3	17	62	-22.8	2.382679865	-	-	-
Abdel-Salam et al., 2014 [15]	46.4	7.3	20	51.7	6.6	23	-5.3	2.670287397	-	-	-
Mansour et al., 2011 [16]	70.8	3.3	30	69	5.2	23	1.8	1.240424191	-	-	-
-	-	-	-	-	-	-	-	-	- 8.69 [-23.06, 5.69]	Chi² = 84.27	p= 0.236
-	-	-	-	-	-	-	-	-	-	df= 2	-
-	-	-	-	-	-	-	-	-	-	P = 0.001	-
-	-	-	-	-	-	-	-	-	-	I² = 97.39	-

Effect of Ivabradine on NT-proBNP

**Figure 7:**
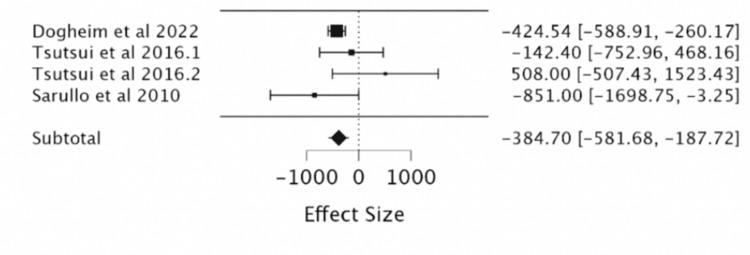
Forest plot of the mean difference in NT-proBNP levels with ivabradine vs. placebo. Sources include: Dogheim et al. (2022) [10], Tsutsui et al. (2016.1) [12], Tsutsui et al. (2016.2) [12], and Sarullo et al. (2010) [14].

The combined result of the three RCTs (a total of 283 patients, with 141 in the ivabradine group and 142 in the placebo group) found that the use of ivabradine was significantly more effective at reducing NT-proBNP levels in patients with heart failure (MD -384; 95%CI -581.68, -187.72) (Table 5, Figure 7).

**Table 5:** Effect of ivabradine vs. placebo on NT-proBNP levels. SD: standard deviation, Iva: ivabradine, NT-proBNP: N-terminal pro b-type natriuretic peptide, df: degree of freedom, P: p-value, I^2^ : Higgins I-squared statistic, CI: confidence interval

Study or subgroup	Mean NT-proBNP Iva		SD NT-proBNP Iva		Total Iva	Mean NT-proBNP placebo	SD NT-proBNP placebo	Total placebo	Mean difference	SD mean difference	Subtotal (95% CI)	Heterogeneity	Test of overall effect Z= -3.82
Dogheim et al., 2022 [10]	728.33	293.37		30		1152.87	353.44	30	-424.54	83.8621469	-	-	-
Tsutsui et al., 2016 [12]	1006.1	1120.5		41		1148.5	1650.2	41	-142.4	311.5140168	-	-	-
Tsutsui et al., 2016 [12]	1656.5	2842.5		40		1148.5	1650.2	41	508	518.0865698	-	-	-
Sarullo et al., 2010 [14]	1434	1273		30		2285	1998	30	-851	432.5325807	-	-	-
-	-	-		-		-	-	-	-	-	-384 [-581.68, -187.72]	Chi² = 4.9	p= 0.001
-	-	-		-		-	-	-	-	-	-	df=3	-
-	-	-		-		-	-	-	-	-	-	P= 0.001	-
-	-	-		-		-	-	-	-	-	-	I² = 6.01	-

Effect of Ivabradine on BNP

**Figure 8:**
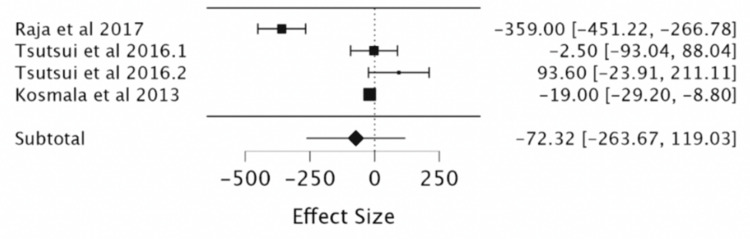
Forest plot of the mean difference in BNP levels with ivabradine vs. placebo. Sources include: Raja et al. (2017) [11], Tsutsui et al. (2016.1) [12], Tsutsui et al. (2016.2) [12], and Kosmala et al. (2013) [13].

Three RCTs (a total of 350 patients, with 174 in the ivabradine group and 175 in the placebo group) enrolled in our analysis that studied the effect of ivabradine on BNP levels, which revealed no significant change (MD -72.32, 95%CI -263.67,119.03) (Table 6, Figure 8).

**Table 6:** Effect of ivabradine vs. placebo on BNP. SD: standard deviation, BNP: brain natriuretic peptide, Iva: ivabradine group, df: degree of freedom, P: p-value, I^2^ : Higgins I-squared statistic, CI: confidence interval

Study or subgroup	Mean BNP Iva	SD BNP Iva	Total Iva	Mean BNP placebo	SD placebo	Total placebo	Mean difference	SD mean difference	Subtotal (95% CI)	Heterogeneity	Test of overall effect Z= -0.741
Raja et al., 2017 [11]	112	58	63	471	366	62	-359	47.05292202	-	-	-
Tsutsui et al., 2016 [12]	181.7	210	41	184.2	208.3	41	-2.5	46.19388884	-	-	-
Tsutsui et al., 2016 [12]	277.8	318.5	40	184.2	208.3	41	93.6	59.95266349	-	-	-
Kosmala et al., 2013 [13]	50	21.3206	30	69	19.2331	31	-19	5.204317024	-	-	-
-	-	-	-	-	-	-	-	-	-72.32 [-263.67,119.03]	Chi² = 53.25	p= 0.236
-	-	-	-	-	-	-	-	-	-	df= 3	-
-	-	-	-	-	-	-	-	-	-	P= 0.001	-
-	-	-	-	-	-	-	-	-	-	I² = 96.85	-

Adverse Events

**Figure 9:**
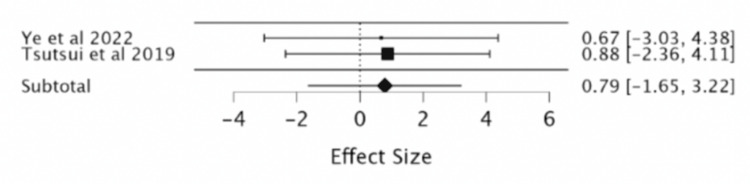
Forest plot of the risk ratio of cardiovascular disease (CVD) mortality with ivabradine vs. placebo. Sources included: Ye et al. (2022) [8], Tsutsui et al. (2019) [9].

CVD mortality: Two RCTs noted no significant reduction in CVD mortality with the use of ivabradine (RR = 0.79; 95% CI -1.65, 3.22) (Table 7, Figure 9).

**Table 7:** Effect of ivabradine vs. placebo on CVD mortality. Iva: ivabradine, CVD: cardiovascular disease, df: degree of freedom, P: p-value, I^2^ : Higgins I-squared statistic, CI: confidence interval

Study or subgroup	Events CVD mortality Iva	Total Iva	Events CVD mortality placebo	Total placebo	Risk ratio	Confidence interval	Subtotal (95% CI)	Heterogeneity	Test of overall effect Z= 0.63
Ye et al., 2022 [8]	4	170	6	172	0.6745	0.1938 to 2.3479	-	-	-
Tsutsui et al., 2019 [9]	7	127	8	127	0.875	0.3270 to 2.3410	-	-	-
-	-	-	-	-	-	-	0.79 [-1.65, 3.22]	Chi² = 53.25	p= 0.52
-	-	-	-	-	-	-	-	df = 3	-
-	-	-	-	-	-	-	-	P = 0.001	-
-	-	-	-	-	-	-	-	I² = 96.85	-

**Figure 10:**
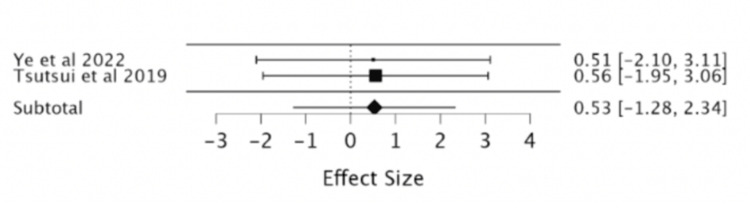
Forest plot of the risk ratio of heart failure (HF) readmission with ivabradine vs. placebo. Sources included: Ye et al. (2022) [8] and Tsutsui et al. (2019) [9].

Heart failure readmission: According to two RCTs evaluated in our study, ivabradine did not significantly reduce the risk of HF readmission (RR = 0.53; 95% CI -1.28, 2.34) (Table 8, Figure 10).

**Table 8:** Effect of ivabradine vs. placebo on HF readmission. HF: heart failure, Iva: ivabradine, df: degree of freedom, P: p-value, I^2^ : Higgins I-squared statistic, CI: confidence interval

Study or subgroup	Events HF readmission Iva	Total Iva	Events HF readmission placebo	Total placebo	Risk ratio	Confidence interval	Subtotal (95% CI)	Heterogeneity	Test of overall effect Z= 0.57
Ye et al., 2022 [8]	16	170	32	172	0.5059	0.2885 to 0.8869	-	-	-
Tsutsui et al., 2019 [9]	20	127	36	127	0.5556	0.3410 to 0.9052	-	-	-
-	-	-	-	-	-	-	0.53 [-1.28, 2.34]	Chi² = 0.0007	p= 0.56
-	-	-	-	-	-	-	-	df=1	-
-	-	-	-	-	-	-	-	P= 0.97	-
-	-	-	-	-	-	-	-	I² = 0	-

**Figure 11:**
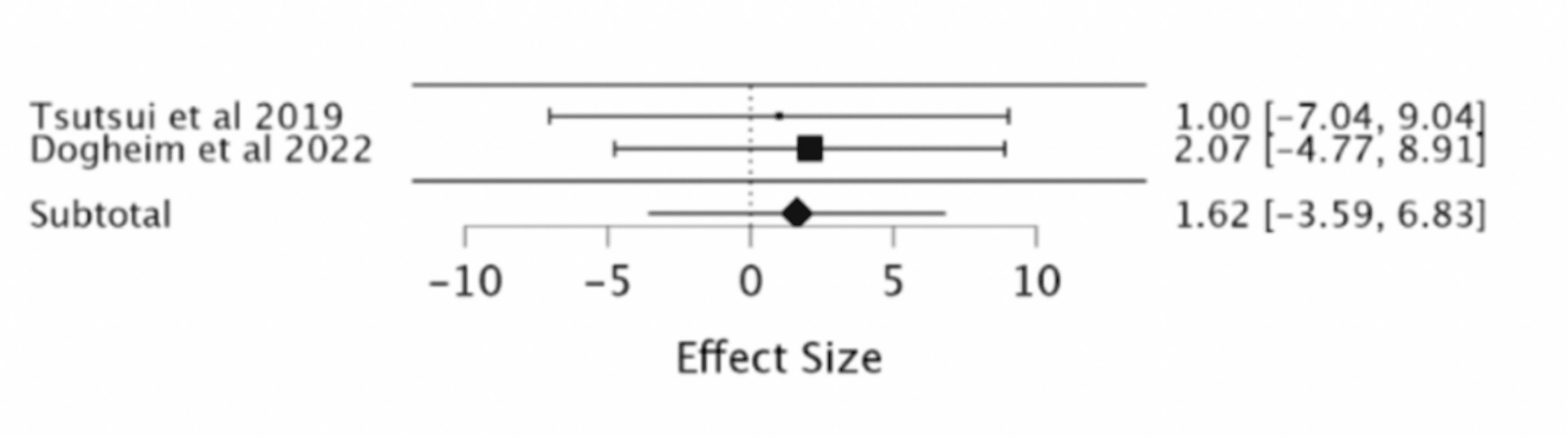
Forest plot of the risk ratio of asymptomatic bradycardia with ivabradine vs. placebo. Sources included: Tsutsui et al. (2019) [9] and Dogheim et al. (2022) [10].

Asymptomatic bradycardia: Two RCTs that were analyzed indicated the reduction in risk of asymptomatic bradycardia with ivabradine treatment was insignificant (RR 1.62; 95% CI -3.59, 6.83) (Table 9, Figure 11).

**Table 9:** Showing the effect of ivabradine vs. placebo on asymptomatic bradycardia. Iva: ivabradine, df: degree of freedom, P: p-value, I^2^ : Higgins I-squared statistic, CI: confidence interval

Study or subgroup	Events asymptomatic bradycardia Iva	Total Iva	Events asymptomatic bradycardia placebo	Total placebo	Risk ratio	Confidence interval	Subtotal (95% CI)	Heterogeneity	Test of overall effect Z = 2.65
Tsutsui et al., 2019 [9]	1	127	1	127	1	0.0632 to 15.8145	-	-	-
Dogheim et al., 2022 [10]	2	30	1	30	2.071	0.178 to 24.148	-	-	-
-	-	-	-	-	-		1.62 [-3.59, 6.83]	Chi² = 0.04	p= 0.52
-	-	-	-	-	-		-	df= 1	-
-	-	-	-	-	-		-	P= 0.84	-
-	-	-	-	-	-		-	I² =0	-

**Figure 12:**
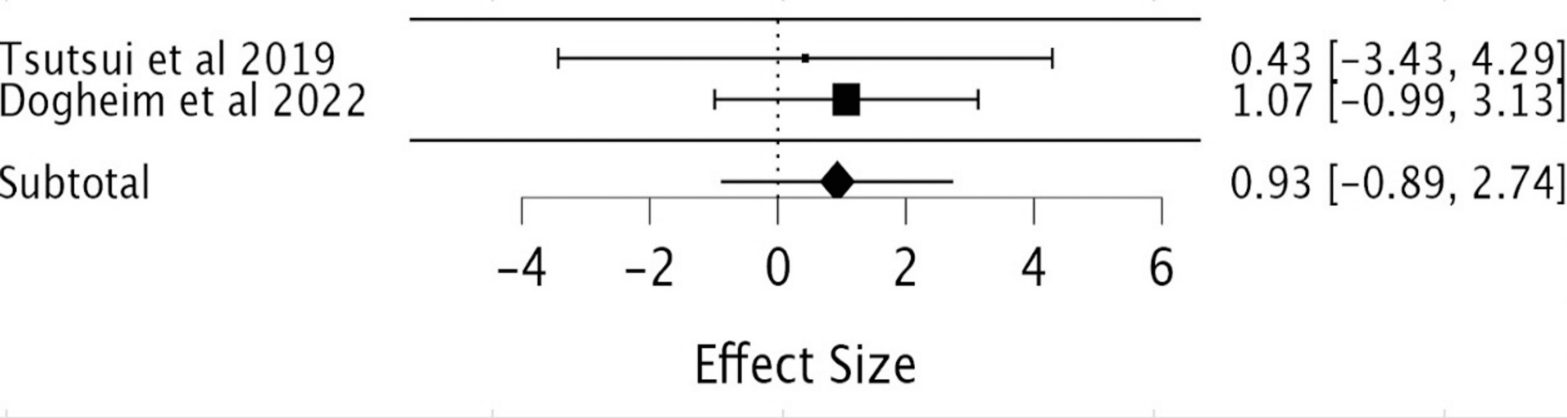
Forest plot showing the risk ratio of atrial fibrillation with ivabradine vs. placebo. Sources included: Tsutsui et al. (2019) [9] and Dogheim et al. (2022) [10].

Atrial fibrillation: Analysis of two RCTs in this study revealed no statistically significant reduction in the risk of atrial fibrillation in patients taking ivabradine (RR = 0.93; 95% CI -0.89, 2.74) (Table 10, Figure 12).

**Table 10:** Effect of ivabradine vs. placebo on atrial fibrillation. Afib: atrial fibrillation, Iva: ivabradine, df: degree of freedom, P: p-value, I^2^ : Higgins I-squared statistic, CI: confidence interval

Study or subgroup	Events Afib Iva	Total Iva	Events Afib placebo	Total placebo	Risk ratio	Confidence interval	Subtotal (95% CI)	Heterogeneity	Test of overall effect Z = 1.002
Tsutsui et al., 2019 [9]	3	127	7	127	0.4286	0.1134 to 1.6204	-	-	-
Dogheim et al., 2022 [10]	2	30	0	30	1.071	0.974 to 1.179	-	-	-
-	-	-	-	-	-	-	0.93 [-0.89, 2.74]	Chi² = 0.083	p= 0.316
-	-	-	-	-	-	-	-	df= 1	-
-	-	-	-	-	-	-	-	P= 0.77	-
-	-	-	-	-	-	-	-	I² = 0	-

Heterogeneity Assessment

Moderate heterogeneity investigation in the resting HR and LVEF was stratified into subgroups based on the follow-up period. Excluding studies with a follow-up duration <3 months demonstrated an HR reduction of -11.34 (-12.84, -9.95; I^2^ = 0.0008; chi = 3.43; df = 3) and an improvement in LVEF of 3.91 (2.59, 5.22; I^2^ 0%; chi = 2.35; df = 4), thus indicating that the follow-up period was a potential source of heterogeneity.

When the HR underwent subgroup analysis according to population age whereby a study done on children was eliminated leaving behind only adults, a pooled HR reduction of -11.58 (-12.77, -10.40) was obtained. Therefore, the results are maintained and age is not a source of heterogeneity.

Furthermore, high heterogeneity was present in outcomes for MLWHF, BNP levels, and CVD mortality, which persisted upon multiple subgroup analyses. For outcomes of exercise HR, six-minute walk test (6MWT), Kingston Caregiver Stress Scale (KCSS) QoL assessment, and symptomatic bradycardia, there were not sufficient studies to meta-analyze, thereby limiting our ability to conclude these outcomes.

Findings

It is already evident that HR has a direct inverse correlation with LVEF, and this study confirmed that ivabradine is effective in its current clinical role in the treatment of heart failure by regulation of both these parameters [1,5,7]. The meta-analysis showed that irrespective of age, ivabradine had a clinically significant reduction in HR and improvement in LVEF in patients [1,8-9]. Pei et al., in 2019, found similar, significant, ivabradine-induced cardiopulmonary function improvements with a reduction in HR and improvement in LVEF. Our study showed a comparable reduction in HR (MD = -11.7, 95% CI: -12.88, -10.51, P < 0.001) to Pei et al. in 2019 (MD = -17.30, 95% CI: 19.52-15.08, P < 0.00001). Our study’s LVEF improvement (MD = 3.03, 95% CI: 2.07, 3.98, P < 0.001) also correlated to theirs (MD = 3.90, 95% CI: 0.40-7.40, P < 0.0001). This was done in a similar study design as well with a meta-analysis of RCTs of corresponding databases such as PubMed, Embase, Cochrane Central Register of Controlled Trials (CENTRAL), and Clinical Trials. Thus, the population is also analogous [17]. In the analysis of secondary outcomes, there is no clear correlation between ivabradine treatment and CVD mortality, QoL using the MLWHF questionnaire, NT-proBNP or BNP, and events such as CVD mortality, HF readmission, or development of atrial fibrillation [10-11]. By contrast, Dogheim et al. (2022) found that ivabradine did have a significant decrease in the levels of biomarkers NT-Pro BNP (p < 0.001) and neopterin (p < 0.001) in the ivabradine group after three months of intervention as compared to baseline [10].

Clinical Relevance

It is well understood that ejection fraction is inversely proportional to HR, with a gradual progressive decrease in ejection fraction occurring as HR increases within a physiological range with statistically significant reductions in ejection fractions occurring with an incremental rise in HR 30 beats per minute or greater [18]. This is relevant to clinical outcomes as it has been previously reported that LVEF is inversely proportional to the mortality rate in patients with abnormal ejection fractions [19]. Rapid resting HR can lead to detrimental effects on left ventricular function and has been associated with negative outcomes in patients with CVDs. Therefore, reducing resting HR to reduce cardiovascular morbidity and mortality is a therapeutic target among drug manufacturers. Ivabradine reduces HR but does not affect myocardial contraction, relaxation, or ventricular repolarization [20]. From the inferred impact ivabradine-induced improvement in cardiovascular function can have on clinical outcomes, healthcare providers may opt in the future for earlier use of ivabradine for targeted therapy in the therapeutic goals of improved LVEF and reduced HR.

Mechanism of Action

Ivabradine is an HR-lowering agent. By specifically blocking the I(f) channel, a mixed sodium-potassium inward channel, it inhibits the cardiac pacemaker current that controls the spontaneous diastolic depolarization in the sinoatrial (SA) node and hence lowers the HR [20].

Heterogeneity and Consistency

In our study, the use of ivabradine showed a significant reduction in HR and increased LVEF compared to the placebo. Subgroup analysis on studies with >3 months follow-up increased this correlation. Therefore, follow-up duration is a potential source of heterogeneity. It can be seen in Zugck et al.'s study in 2014 that follow-up duration can have a significant correlation with the efficacy ivabradine can have on HR reduction. In the prospective, open-label multicenter INTENSIFY study, the mean HR of patients was reduced by ivabradine from 85 ± 11.8 bpm at baseline to 72 ± 9.9 bpm after one month and 67 ± 8.9 bpm after four months, emphasizing the possible correlation of improved results with duration of treatment [21]. Subgroup analysis according to age maintained the initial pooled results; thus, age is not a source of heterogeneity. Bohm et al. studied the effect of ivabradine in HF patients with HR <75 bpm and >75 bpm and found similar results to our study with a significant reduction in HR. However, in contrast to our study, they were able to stratify the results based on age and with results showing a greater effect on the HR >75 beats per minute (bpm) group that comprised younger patients with other factors to consider (smokers, lower LVEF, higher New York Heart Association (NYHA) classification, and non-ischemic HF), suggesting that certain age groups may benefit more [22].

Implications for future research

Ivabradine is indicated to reduce the risk of hospitalization for worsening HF in patients with stable, symptomatic chronic HF with an LVEF of 35% or less, who are in sinus rhythm with a resting HR of 70 bpm or greater, and are either receiving maximally tolerated doses of beta-blockers or have a contraindication to beta-blocker use. Future studies should consider larger sample sizes and longer follow-up periods to better elucidate the potential impact of ivabradine on patient outcomes across various cardiovascular conditions. Moreover, future studies can look at the efficacy of the drug over time, as well as based on age, since previous studies would have found a correlation with improved cardiovascular function over longer follow-up periods and in younger age groups. This can be further investigated for more definitive evidence to guide therapeutic use indications and guidelines. In addition, with the statistically significant reduction in HR and increase in ejection fraction, exploring its effects on different patient subgroups may provide insights into personalized treatment strategies in clinical practice.

Limitations

Due to the extensive exclusion criteria, the number of RCTs included in the meta-analysis was relatively small, which limits the generalization of the results of the study. Restricting the search to English-language studies may potentially exclude relevant non-English studies that could contribute additional data or different perspectives on ivabradine's effects. Even within this meta-analysis, there may be variability within the RCTs regarding patient characteristics (e.g., age, comorbidities) and study designs (e.g., dosages, treatment durations), which could influence the interpretation and applicability of the findings across different patient groups. Despite the rigorous inclusion criteria, individual studies within the meta-analysis may still carry biases (e.g., selection bias, performance bias) that could affect the overall robustness and reliability of the pooled results. Subgroup analysis of longer follow-up periods resulted in greater significance. From this, a potential limitation would be the inclusion of studies with relatively short follow-up periods, potentially restricting the comprehensive evaluation of ivabradine's long-term effect. Another limitation of this meta-analysis was its restriction to studies with ECHO results as an inclusion criterion, potentially excluding relevant studies that may strengthen other outcomes assessed such as HR without ejection fraction. Addressing these limitations in future research could strengthen the validity and applicability of our findings. The usage of additional treatment in the placebo group or the addition of ivabradine as an add-on therapy could also be another limiting factor for the study. It should also be noted that the randomized trials are compared to current best practices for heart failure with reduced ejection fraction management, which is combined medical therapy, as a control group, and that ivabradine would not be used as a single agent as an intervention, so there might be study heterogeneity depending on exact drug combinations used.

Final thoughts

This meta-analysis consolidates current evidence on the impact of ivabradine therapy on cardiovascular parameters in managing heart failure. The findings indicate a significant reduction in HR accompanied by an improvement in ejection fraction, suggesting a beneficial role in enhancing cardiac function through HR modulation. In summary, while ivabradine demonstrates clear efficacy in reducing HR and improving ejection fraction, its overall influence on broader clinical endpoints remains inconclusive based on current evidence. Continued research efforts on the comparison of ivabradine to other standard therapies are also crucial to better understand the comprehensive therapeutic profile and optimal utilization of ivabradine in cardiovascular management.

## Conclusions

The main outcomes of this meta-analysis were HR and LVEF. It can be concluded with moderate certainty that ivabradine significantly reduced HR and improved LVEF in patients with heart failure with reduced ejection fractions, irrespective of age. Ivabradine is effective in reducing cardiovascular morbidity by reducing resting HR. In addition, ivabradine was shown to have no impact on mortality and readmission rates in patients with heart failure (weak certainty based on the number of studies yielded). Future research should be conducted in larger, multicenter studies that may have more diverse patient populations and longer follow-up periods to elucidate further the impact of ivabradine on patient outcomes across various cardiovascular conditions.
